# Synthesis, structural, optical, and thermal properties of LaFeO_3_/Poly(methyl methacrylate)/Poly(vinyl acetate) nanocomposites for radiation shielding

**DOI:** 10.1038/s41598-024-54207-5

**Published:** 2024-02-14

**Authors:** M. Khalifa, Adel M. El Sayed, Said M. Kassem, E. Tarek

**Affiliations:** 1 Physics Department, Faculty of Science, Fayoum University, El Fayoum, 63514 Egypt; 2 Faculty of Computers and Information Technology, National Egyptian E-Learning University, Giza, 12611 Egypt; 3 Radiation Protection and Dosimetry Department, National Center for Radiation Research and Technology (NCRRT), Egyptian Atomic Energy Authority (EAEA), Cairo, Egypt

**Keywords:** LaFeO_3_, PMMA/PVAc Nanocomposites, Co-precipitation, Dual bandgap materials, Thermal stability, γ-ray shielding, Phy-X/PSD software, Materials science, Nanoscience and technology, Optics and photonics, Physics

## Abstract

This work is an attempt to develop flexible radiation shielding based on a blend of polymethyl methacrylate (PMMA)/polyvinyl acetate (PVAc) and LaFeO_3_ nanoparticles (NPs). LaFeO_3_ and LaFeO_3_/PMMA/PVAc were made using simple chemical techniques. A high-resolution transmission electron microscope (HR-TEM) and X-ray diffraction (XRD) showed that well-crystallized LaFeO_3_ NPs with particles 79 nm in size and an orthorhombic shape were obtained. In addition, XRD confirmed the existence of PMMA, PVAc, and LaFeO_3_ in the nanocomposite films. Fourier transform infrared (FTIR) confirmed that the LaFeO_3_ NPs and the reactive functional groups in the blend interacted with each other. Field emission-scan electron microscope (FE-SEM) analysis showed that PMMA and PVAc form a homogenous blend and that the LaFeO_3_ NPs were spread out inside and on the blend surface. The samples showed transmittance in the range of 30–74% and a small extinction coefficient (≤ 0.08). The samples exhibited a dual-band gap structure, and the direct (indirect) band gap shrank from 5.1 to 4.7 eV (4.9 to 4.4 eV). The thermal analyses showed that the samples are thermally stable up to 260 °C. The Phy-X/PSD software was used to figure out the theoretical gamma-ray attenuation parameters, such as the mass attenuation coefficient, the mean free path, and the half-value layer, for different PMMA/PVAc + x% LaFeO_3_ composites. It is demonstrated that the PMMA/PVAc + 10 wt% LaFeO_3_ sample exhibits much better shielding effectiveness than PMMA/PVAc, and hence it is suitable for protecting against radiation.

## Introduction

Searching for advanced radiation shielding materials became essential in various fields, such as space exploration, nuclear medical imaging, and nuclear waste storage. Exposure to γ-rays is biologically hazardous and can lead to cell mutation, organ damage, and other unwanted effects^1^. To prevent the energy transfer from these highly energetic photons to the electrons, materials with a high atomic number, such as lead compounds, should be used. However, due to the toxicity of Pb, many attempts have been made to fabricate and use other materials based on bioglasses and polymeric compounds^2,3^. The development of polymeric blends reinforced with nano-sized materials has gained increasing attention worldwide. Polymer nanocomposites are multifunctional materials that can be applied for catalytic reduction of pollutants, environmental applications, food backing, and various medical and pharmaceutical purposes^4–6^. Moreover, when compared to some alloys and bioglasses used in space applications^7–10^, these polymer nanocomposite materials can shield radiation well while having lower mass and density, being easy to handle, being flexible, and producing less secondary radiation.

Poly(methyl methacrylate), PMMA, also known as acrylic, is a stiff polymer that exhibits interesting hardness, optical clarity, and abrasion resistance. As a result, it is used a lot in micro-photonics, optical lenses and devices, solar cells instead of glass, dosimetry, alpha particle detection, and as a base for nanofillers to block gamma-rays and UV light^9^. Cao et al.^1^ reported that the PMMA mixed with 15.6–44 wt% Bi_2_O_3_ particles showed good blocking γ-rays with energies up to 1 MeV. In addition, MWCNTs/PMMA was 18% lighter in mass than Al for stopping the protons with the same energy^7^. According to Soni et al.^8^, the dispersion of ZnO/SiO_2_ NPs inside PMMA increases the gamma radiation shielding. Putting MWCNTs inside poly(methyl methacrylate), or PMMA, made it more stable at high temperatures and cut down on the production of neutrons in pure PMMA. Saudi et al.^10^ mixed PMMA with Zn, Hg, or Cd carbazone to block γ-rays with energies between 662 and 1333 keV. Among them, the Hg/PMMA complex exhibited the best gamma shielding capacity for medical radiation shielding. According to Bel et al.^11^, adding 5–40 wt% colemanite (Ca_2_B_6_O_11_·5H_2_O) to PMMA made it 11.1% better at blocking γ-rays from Cs-137 and 38.56% better at blocking neutrons.

Poly(vinyl acetate), or PVAc, known as white glue, is a cheap thermoplastic biodegradable polymer. Due to its mechanical and thermal properties and low melting temperature (~ 30 °C), it is extensively used in the furniture and packaging industries and for the electromagnetic interference shielding^12–14^. Due to the existence of the COOH group in its structure, PVAc is easy to form into biocompatible and safe films for use in biomedical purposes^15,16^. PVAc and PMMA form a pair of polymers that are essential for the blending technology. Different physical properties of the PMMA/PVAc blend and PMMA/PVAc nanocomposites were reported. This blend's optical properties and thermal stability were improved by adding fullerene C_60_^17,18^. It can now be used in solar concentrators. Modifying PMMA (30%)/PVAc (70%) by mixing with ethylene carbonate and loading TiO_2_ nanofiller improved its electrical conductivity by one order of magnitude^19^.

On the other hand, the ABO_3_ perovskite bimetallic oxides are attracting the attention of researchers worldwide due to their unlimited uses and energy applications, such as for solid fuel cell electrodes, solid-state and rechargeable batteries, hard drives, read heads in advanced computers, automotive exhaust catalysts, solid oxide fuel cell electrodes, gas sensing, efficient hydrogen conversion and storage, and optoelectronic devices^20,21^. Bulk LaFeO_3_ is a perovskite that is antiferromagnetic semiconductor. Fe cations occupy the center of the octahedrons, O on the corners, and La ions occupy 12-coordinated sites surrounding the octahedrons, contributing to their structural stability. Therefore, LaFeO_3_ exhibits excellent thermal stability, high electron mobility, and gas-sensing ability^22,23^. These researchers^24^ made LaFeO_3_ nanotubes with a diameter of about 200 nm. They discovered that raising the calcination temperature from 600 to 800 °C makes the tube surfaces denser and rougher and makes them more sensitive to *n*-butanol. Tsai and Su created crystalline and amorphous LaFeO_3_ NPs doped with sea-urchin-like Au that has a band gap that can be changed from 2.09 to 2.29 eV so that hydrogen can be made by splitting water^25^. LaFeO_3_ and (50–70 wt%) LaFeO_3_/poly(vinylidene) fluoride, PVDF, were made by a chemical pyrophoric reaction and solution casting, respectively, by Nath et al.^26^. The magnetic and electric properties found suggested that 50 wt% LaFeO_3_/PVDF could be used for smart energy storage, energy harvesting, and magnetoelectric devices. Kum-once and Thongbai^27^ prepared LaFeO_3_ particles of average size 194 nm on the combustion route and used them for improving the dielectric properties of PVDF, where the dielectric permittivity of 0.5 wt% LaFeO_3_/PVDF was five times higher than that of PVDF, and the dielectric loss tangent decreased by 0.059.

The literature review shows that no one has yet written about how LaFeO_3_ nano-perovskite affects the physical properties of the PMMA/PVAc blend. This is the first study that the authors know of that looks at how co-precipitated LaFeO_3_ NPs change the structure, optical, and thermal properties of a blend of PMMA and PVAc. The aim is to design novel flexible, cost-effective, and sustainable thermoplastic radiation shielding materials composed of LaFeO_3_/PMMA/PVAc for γ-rays with beneficial gamma-ray shielding features and improved capacity for use in the various fields of medicine and industry. LaFeO_3_ NPs and LaFeO_3_/PMMA/PVAc nanocomposites were prepared by co-precipitation and solution casting, which are facile, cost-effective, environmentally friendly methods, and allow rapid and large-scale synthesis^22^.

## Experimental procedures

### Materials and preparation

In the co-precipitation process, equal amounts of lanthanum nitrate hexahydrate [La(NO_3_)_3_.6H_2_O, molecular weight (*M*_W_) = 433 g/mol, IndiaMart, India] and ferric nitrate nonahydrate [(Fe(NO_3_)_3_.9H_2_O, *M*_W_ = 403.95 g/mol, Merck, Germany] were dissolved in 100 ml of double-distilled (DD) water by using a magnetic stirrer for 30 min. Then a 0.5 M NaOH solution was added dropwise until a brown precipitate was formed. The precipitate was collected, washed several times using DD water, and dried at 100 °C for 1.0 h. Then, it was calcined in an air furnace at 900 °C for 2.0 h and cooled to room temperature (RT) naturally. In an earlier report, it was said that the purest LaMO_3_ NPs (M = Cr, Co, Ni, Fe, or Mn) can be made at a calcination temperature of 900 in the air^21^.

The blend film was prepared by solution casting, where ~ 0.9 g of PVAc [[CH_2_CH(O_2_CCH_3_)]_n_, *M*_W_ = 10^5^ g/mol, Alpha Chemika, India) and 0.3 g of PMMA [[CH_2_C(CH_3_)(CO_2_CH_3_)]_n_, *M*_W_ = 1.2 × 10^4^ g/mol, Acros Organics, UK) were dissolved in 60 ml of tetrahydrofuran (THF) [(CH_2_)_4_O, *M*_W_ = 72.1 g/mol, Aldrich, Germany]. This made the PMMA/PVAc blend. The polymers (in the form of powder) and THF were put in a tightly closed beaker and stirred for 1.0 h at 40–45 °C to get a homogenous and clear solution. The blended solution is cast into pre-cleaned glass Petri dishes. The same steps were used to make a LaFeO_3_/PMMA/PVAc nanocomposite. Then, 0.5, 1.0, 3.0, 6.0, and 10 wt% of LaFeO_3_ were dissolved in 20 ml of THF using ultrasound and added to the blend solution. The mass of the LaFeO_3_ as fillers (${w}_{{\text{LaFe}}}$) was determined using the following equation: $x \left(wt\%\right)= \frac{{w}_{{\text{LaFe}}} \times 100}{{w}_{{\text{LaFe}}}+ 1.2}$ where *x* = 0.5–10 and the "1.2" in the denominator is the mass of the PMMA/PVAc blend. The dishes were left on a leveled plate in the air to allow for the THF solvent to evaporate slowly. Finally, care was taken during the removal of the film to obtain self-standing films with uniform thickness.

Using the following equation, the Archimedes rule was applied to determine the density of water-soaked LaFeO_3_/PMMA/PVAc composites:

1 $$\rho =\frac{{W}_{a}}{({W}_{a}-{W}_{w})} {\rho }_{w},$$

where *W*_a_, *W*_w_ and ρ_w_ represent the weight of the LaFeO_3_/PMMA/PVAc composite film in the air, in water, and the water density.

### Characterization techniques

A transmission electron microscope (TEM) of high resolution from JEM 2100, Jeol, Japan, was utilized to investigate the morphology, shape, size, and inter-planer spacing of the co-precipitated LaFeO_3_. A drop of the LaFeO_3_ suspension was placed onto a C-coated Cu grid that was then dried in the air before being transferred to the microscope, which operated at 200 kV. The X-ray diffraction analyses were done for LaFeO_3_ and LaFeO_3_/PMMA/PVAc by using PANalytical X’Pert PRO, Germany, with a Cu K_*α*_ line of wavelength *λ* = 0.1541 nm, in the 2θ range of 5.0–85°. The modes of vibration of the functional groups of the prepared samples were analyzed in the wavenumber range of 400–4000 cm^−1^, at RT, using a Fourier transform infrared (FTIR)/attenuated total reflection (ATR) spectrometer, VERTEX 70/70v, from Bruker Corporation, Germany. The surface morphology of the films, the cross-sectional, thickness determination, and the elemental mapping were performed using FE-SEM (Inspect S, FEI, Holland), coupled with EDAX. A JASCO 630 spectrophotometer from Japan was used to study the transmittance spectra in the UV, Vis, and NIR ranges, covering wavelengths from 200 to 1500 nm. The measurements were carried out at RT. The TGA–DSC thermograms were recorded in the temperature range of 30–650 ℃ using the Perkin Elmer STA 6000, Germany, in an N_2_ atmosphere.

### Radiation attenuating parameters

Phy-X/PSD results from an extensive effort recently completed by Sakar and his team^28^ to create an easy-to-use program that can produce various radiation protection factors. It is accessible online at (https://phy-x.net/PSD) and may help researchers and shielding engineers investigate and provide data on photon attenuation using various systems. Additionally, using NIST's WinXCOM computer program, the mass attenuation coefficients have been compared to their theoretical values by Phy-X/PSD^29,30^. Calculating the mass attenuation coefficient MAC (µ_m_) entails dividing the linear attenuation coefficient (µ) by $\rho$ of the PMMA/PVAc + x% LaFeO_3_ nanocomposite films^28^;

2 $${\mu }_{m}=\left(\frac{\mu }{\rho }\right)=\sum_{i}{W}_{i }\left(\frac{\mu }{\rho }\right)i ,$$

in which W_i_ symbolizes the weight fraction of the ith element in the sample.

The half-value layer (HVL) defines the sample's thickness, decreasing the radiation's intensity by half. The tenth-value layer (TVL) refers to the mean amount of substance required to attenuate 90% of the entire radiation, resulting in a reduction of the initial intensity to one-tenth of its original value. Furthermore, the mean free path (MFP) is the average distance a photon travels between two subsequent interactions. The HVL, TVL, MFP, the total atomic cross section (σ_t,a_), the total electronic cross section (σ_t,e_), the effective atomic number (*Z*_eff_), and the effective electron number (*N*_eff_) are provided in the formulae ((3)–(9))^30,31^;

3 $$HVL={\text{ln}}\left(2\right)/\mu ,$$

4 $$TVL={\text{ln}}(10)/\mu ,$$

5 $$MFP=1/\mu ,$$

6 $${\sigma }_{t,a}={\mu }_{m} \frac{M}{{N}_{A}} ,$$

7 $${\sigma }_{t,e}=\left(\frac{1}{{N}_{A}}\right)\sum_{{\text{i}}}\left(\frac{{f}_{i}{A}_{i}}{{Z}_{i}}\right) {({\mu }_{m})}_{i} ,$$

8 $${Z}_{eff}= \frac{\sum_{i}{f}_{i}{A}_{i}(LAC)}{\sum_{j}\left(\frac{{f}_{j}{A}_{j}}{{Z}_{j}}\right)(LAC)} ,$$

9 $${N}_{eff}=\frac{{N}_{A}}{M} {Z}_{eff} {\sum }_{{\text{i}}}{{\text{n}}}_{{\text{i}}} .$$

## Results and discussion

### Materials morphology and structure

**Figure 1:**
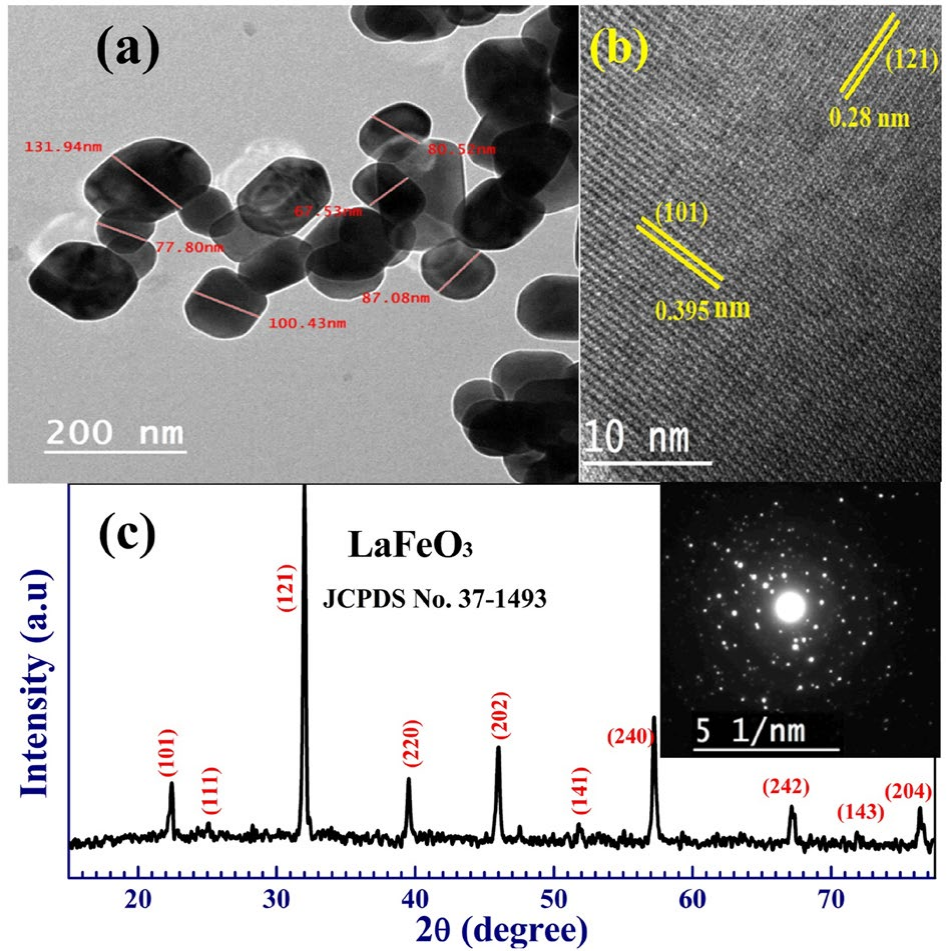
(**a**) HR-TEM, (**b**) d-spacing, and (**c**) XRD pattern of LaFeO_3_ NPs prepared by co-precipitation method. The inset of (**c**) is the selected area electron diffraction (SEAD) for a single particle.

HR-TEM and XRD were employed to obtain a detailed analysis of the LaFeO_3_ microstructure. According to Fig. 1a, the LaFeO_3_ particles prepared by the co-precipitation method have the same shape and size, ranging from 68 to 132 nm, with a mean of 79 nm and a standard deviation of 8.3 nm. This value is smaller than the particle size of 194 nm for LaFeO_3_ prepared by the combustion method^27^. In addition, LaFeO_3_ NPs prepared by a microwave irradiating using the ethylenediamine as a chelating agent were of 45 nm in size, and clusters larger than 100 nm in size^23^. Figure 1b shows clear inter-planar spacing of 0.281 and 0.395 nm, which are assigned to the LaFeO_3_ (1 2 1) and (1 0 1) planes, respectively.

Figure 1c displays the XRD pattern of LaFeO_3_. The diffraction peaks at 2θ = 22.48°, 32.2°, 39.6°, 46°, 57.2°, 67.3°, and 76.5° are corresponding to (1 0 1), (1 2 1), (2 2 0), (2 0 2), (2 4 0), (2 4 2), and (2 0 4) crystal planes. Besides, there are some peaks of very low intensity at about 25° (1 1 1), 51.8° (1 4 1), and 71.9° (1 4 3). These peaks and their Miller's indices belong to LaFeO_3_ with an orthorhombic structure and lattice constants of *a* = 0.557 nm, *b* = 0.785 nm, and *c* = 0.555 nm, which is consistent with JCPDS No. 37–1493^22^. Similar results were reported for LaFeO_3_ nanotubes and NPs synthesized by the electrostatic spinning method and microwave irradiation^23,24^. The selected area of electron diffraction that HR-TEM checked is Fig. 1c. In the XRD spectrum, the white spots are caused by electrons being diffracted by the LaFeO_3_ plane. No peaks related to any other phase like Fe_2_O_3_, La_2_O_3_, or their hydroxides are present, indicating the high purity of the product. LaFeO_3_ that had Au added to it was made using high temperatures, and its XRD patterns showed some peaks related to La_2_O_3_ and La(OH)_2_^25^. This result illustrates the high purity of LaFeO_3_ NPs prepared by the co-precipitation method.

**Figure 2:**
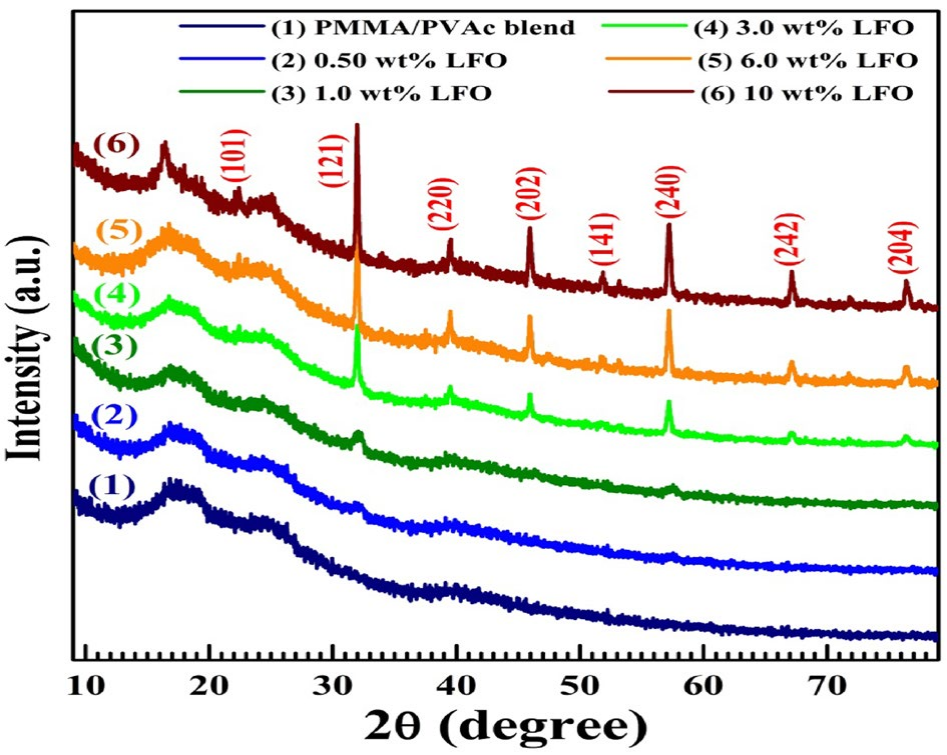
XRD patterns of PMMA/PVAc blend and LaFeO_3_/blend nanocomposites.

The PMMA/PVAc blend's XRD patterns show a broad hump that ranges from 2θ = 15° to 27°, as seen in Fig. 2. This hump is seen as two adjacent peaks centered at 2θ = 18.5 and 25°. The fact that these two peaks are submerged could be because of the non-covalent interactions between PMMA and PVAc^32^. Another hump around 40° depicts the wrong or short-range ordering inside the blend. The two peaks 18.5° and 40° are assigned to PMMA^33^, whereas the broad peak at 2θ = 25° belongs to the amorphous PVAc^15^. Bardak et al. reported that PVAc is only partly crystalline, where two crystalline peaks at 12.5° and ~ 21° were found in its XRD pattern^13^. In addition, Saudi et al.^10^ also found two peaks at ~ 26 and 48 in the XRD pattern of PMMA. When more LaFeO_3_ (0.5–10 wt%) was loaded, the intensity of these peaks went down without moving their centers, which shows that the matrix became more disorganized. In addition, several diffraction peaks related to the LaFeO_3_ fillers appear with increasing intensity as the LaFeO_3_ content has increased. Similar notes were reported in Ref.^11^ for PMMA loaded with colemanite.

**Figure 3:**
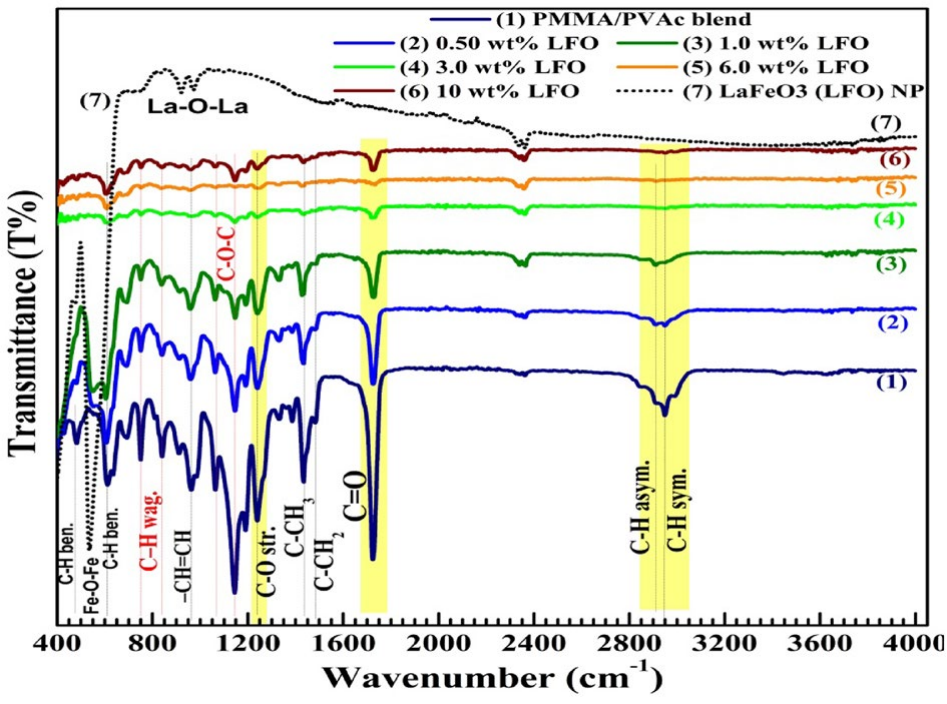
FTIR spectra of PMMA/PVAc blend, LaFeO_3_/PMMA/PVAc nanocomposites and LaFeO_3_.

FTIR is a cool way to investigate the vibrational modes in the materials, how the functional groups in the mix interact with each other and bind to the nanofillers. The FTIR spectra of all samples are presented in Fig. 3. The FTIR spectrum of LaFeO_3_ displays a sharp and deep absorption band at 548 cm^–1^ is assigned to the vibration of Fe–O and Fe–O–Fe bonds^22^. In general, the M–O–M antisymmetric stretching vibrations in the MO_6_ octahedron groups of the ABO_3_ perovskites appear in the energy range 500–700 cm^–1^^20,21^. The two peaks at 920 and 970 cm^–1^ may originate from La–O–La vibrations^34^. The small peaks at about 2330 and 2360 cm^–1^ could be assigned to the symmetric and asymmetric stretching vibrations of CO_2_ adsorbed at the LaFeO_3_ surface^35^.

As seen in Fig. 3, the spectrum of the PMMA/PVAc blend displays a peak at 2950 cm^–1^ and a very small peak at 2900 cm^–1^ which are attributed to the symmetric and asymmetric stretching vibrations of C–H, respectively, in the methyl (–CH_3_) and methylene (=CH_2_) groups^32,36^. The sharp absorption band at 1720 cm^–1^ is attributed to the stretching vibrations of C=O (of the carbonyl group), while the peak at 1440 cm^–1^ and the tiny one at 1480 cm^–1^ are assigned the asymmetric bending vibration of C–CH_3_ and C–CH_2_, respectively^11,37^. Additionally, the band at 1245 cm^–1^ is owing to the stretching vibration of C–O (of the ester group)^37^. The strong bands at 1060 and 1140 cm^–1^ are attributed to the vibration of the C–O–C group^36–38^. The bands at 965 cm^–1^, 842 cm^–1^, 752 cm^–1^ are owing to the vibration of the –CH=CH group, waging vibration and deformation of C–H in CH_3_ groups^11,39^. Finally, C–H bending appears at 610 and 480 cm^–1^^40^. Adding LaFeO_3_ did not create noticeable changes in the peak positions. However, the intensities of the peaks became much weaker as the LaFeO_3_ content increased to 6.0 wt%. This reflects the strong bonding and the deep interactions of the added filler with the blend's functional groups. The nano-sized fillers have a high surface energy and tend to agglomerate with each other when their level reaches 10 wt%. This may make it easier for the blend's functional groups to move around. No new bands are seen in the FTIR spectra of LaFeO_3_/PMMA/PVAc, which suggests that the interactions between these fillers and the blend are physical, *e.g.*, based on Van der Waals force and hydrogen bonding^41^.

**Figure 4:**
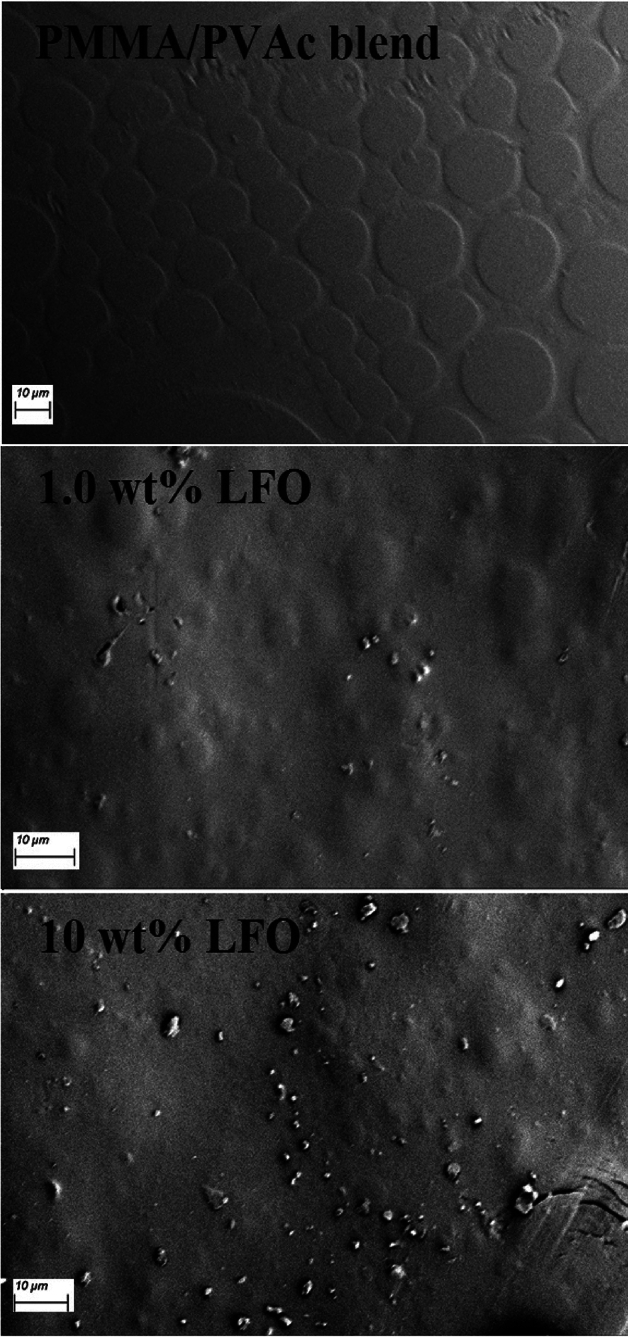
FE-SEM surface images for pure PMMA/PVAc and blend loaded with 1.0 and 10 wt%LaFeO_3_.

**Figure 5:**
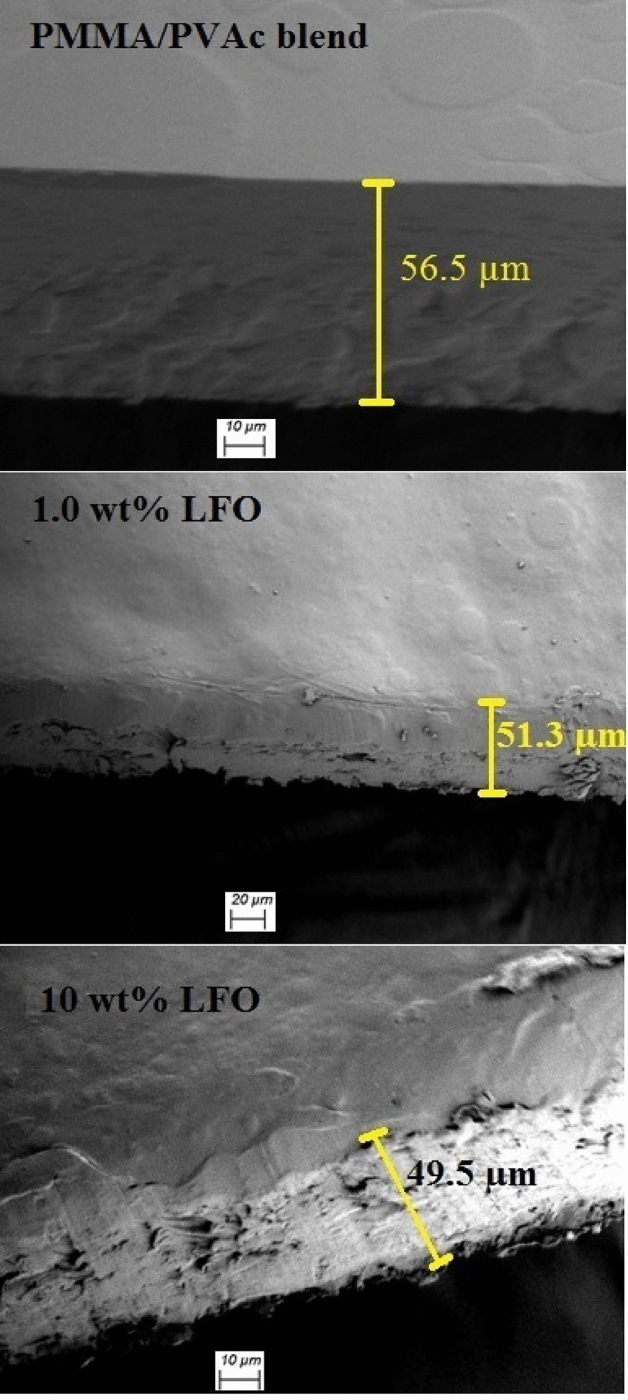
Cross-sectional investigation for PMMA/PVAc and blend loaded with 1.0 and 10 wt%LaFeO_3_.

**Figure 6:**
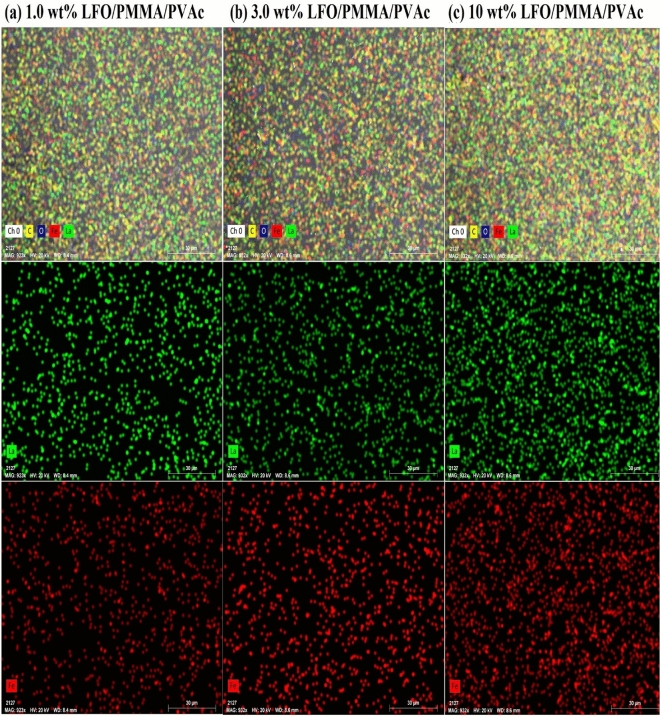
Elemental mapping for PMMA/PVAc loaded with (a) 1.0, (b) 3.0 and (c) 10 wt%LaFeO_3_.

To learn more about the structure and other features of the materials that were made, the shape of the blend's surface and the way the filler was distributed on the surface and inside the blend were studied, as shown in Figs. 4 and 5. The pure PMMA/PVAc blend displays a uniform and homogenous morphology in the form of separated circles. Besides, the surface is nonporous and crack-free. This indicates the good miscibility and compatibility between PVAc and PMMA. This circle structure may be formed due to the interactions between the PMMA and PVAc molecules or during the evaporation of the solvent (THF), which is very volatile. This structure was destroyed gradually with the increase in LaFeO_3_ content. This confirms the interaction between the blend chains and the added nanofillers. Moreover, the nanofillers are aggregated when their content reaches 10 wt% and some cracks appear. Figure 5 is the cross-sectional investigation for the films and illustrates that the PMMA/PVAc blend is nonporous. However, the cracks and voids are created by increasing the filler content. The films' thickness is in the range of 49.5–56.5 µm. Figure 6a–c shows the elemental mapping for the blend loaded with 1.0, 3.0, and 10 wt% LaFeO_3_ nanofillers. The main components of the films are carbon and oxygen. Also, the La and Fe atoms are spread out evenly on the film's surface, and the concentrations of these atoms rise as the LaFeO_3_ content rises. These results confirm the successful preparation of the nanocomposite films with uniform filler distribution.

### Optical characterization

**Figure 7:**
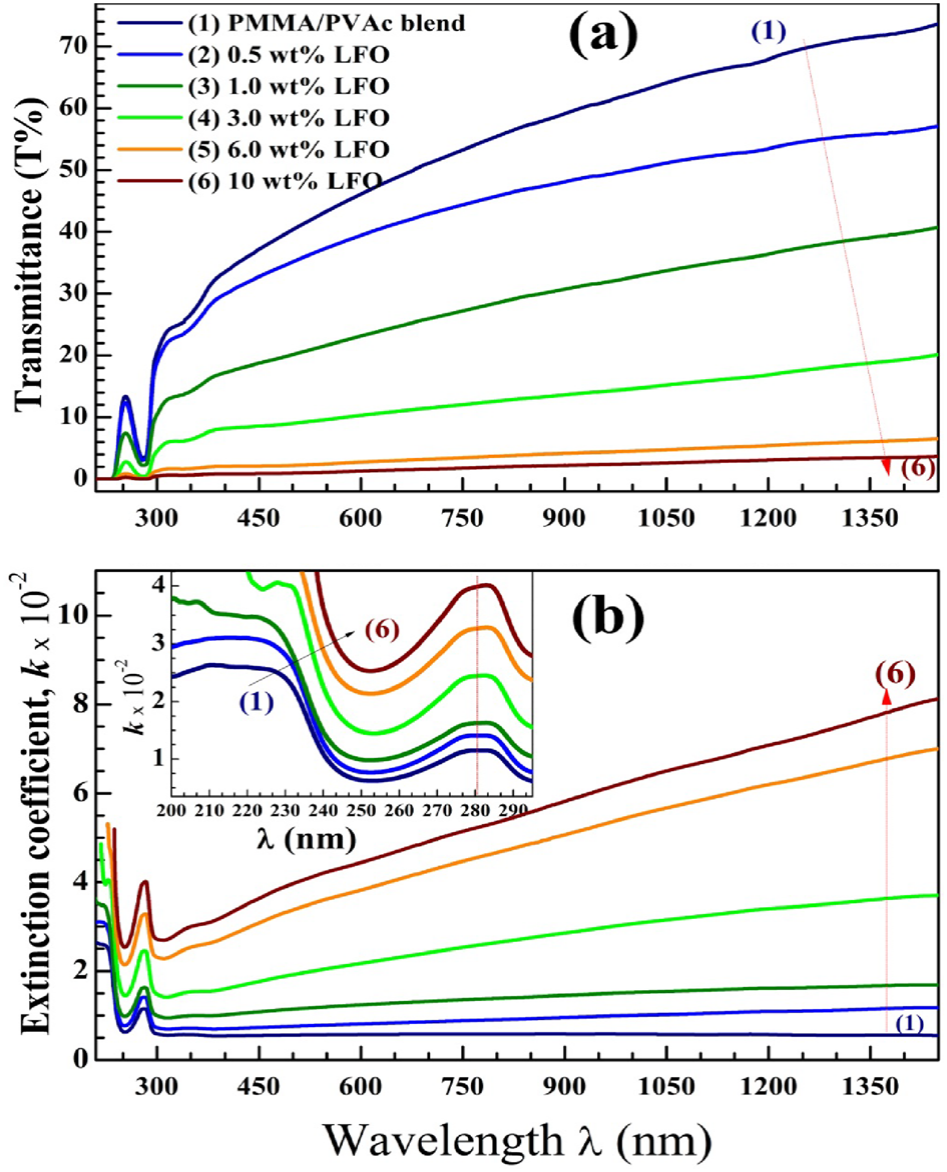
(**a**) transmittance (T%) spectra and (**b**) extinction coefficient (*k*) for PMMA/PVAc blend and LaFeO_3_/blend nanocomposites.

Studying the optical features in the UV–vis-NIR regions and evaluating the band structure is essential to finding suitable optical and optoelectronic applications. Figure 7a,b shows the transmittance (T%) spectra and the dependence of the extinction coefficient *k* on the incident wavelength (*λ*), (where $k=\frac{\lambda \alpha }{4\pi }$ and $\alpha \left(\mathrm{the \,absorption \,coefficient}\right)=\frac{\mathrm{absorption }}{\mathrm{film \,thickness}}$). In the vis–NIR region, the PMMA/PVAc blend has T% values in the range of 30–74%, which is suitable for a wide range of applications. However, this range continuously decreased as the added filler content increased. This is due to the large portion of absorbed and scattered photons by the added LaFeO_3_ NPs. The *k* values decrease in the UV region until *λ* = 250 nm. These highly energetic photons can transport electrons to exited levels without energy loss. The band around 280 nm is assigned to π → π* electronic transition due to the existence of unsaturated bonds (C=O). As seen in the inset of Fig. 7b, the position of this band is shifted to a higher λ value after doping. In the visible and IR regions, the* k* value of the PMMA/PVAc blend is small and constant. Incorporation of LaFeO_3_ with an increased ratio increases the k due to the increase of charge carriers and defect states created after loading LaFeO_3_. In addition, in the highly doped films (3.0–10 wt%), the *k* values increase linearly with *λ*. This suggests the usefulness of these films for sensing applications in the vis–NIR region.

**Figure 8:**
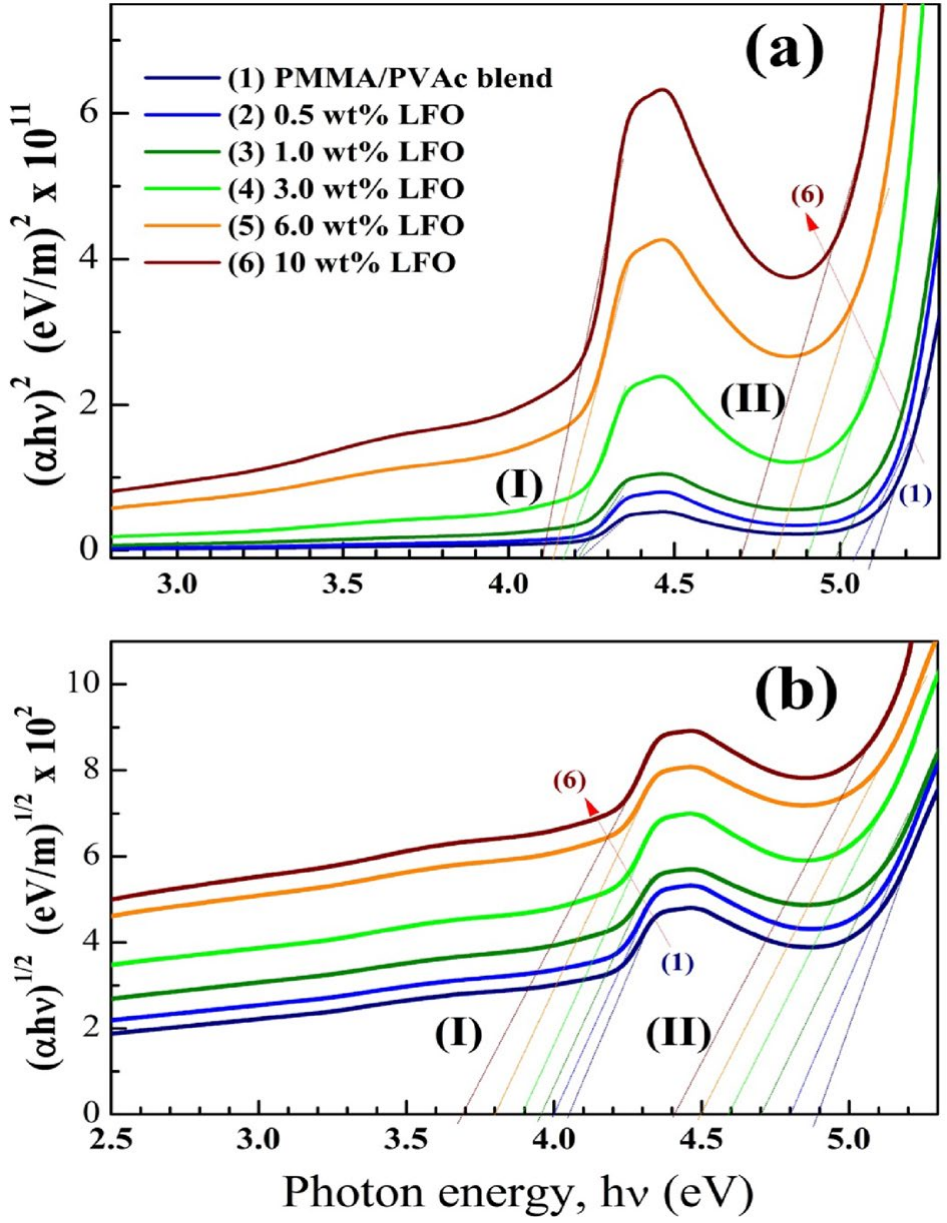
(**a**) Direct and (**b**) indirect optical band gap for PMMA/PVAc blend and LaFeO_3_/blend nanocomposites.

According to Mott and Davis, the direct (${E}_{{\text{gd}}})$ and indirect (${E}_{{\text{gi}}}$) band gaps can be determined by considering the following dependence of $\alpha$ on the photon energy $h\upsilon$: $(\alpha \cdot h\upsilon {)}^{x}={\text{X}}\left(h\upsilon -{E}_{g}\right),$ where *X* is a constant called the band tailing parameter, $h\upsilon ({\text{eV}})=\frac{1242}{\lambda ({\text{nm}})}$, and *x* = 2 or 1/2 based on the type of transition, direct or indirect. Figure 8a,b depicts $(\alpha h\upsilon {)}^{2}$ vs. $h\upsilon$ and $(\alpha h\upsilon {)}^{1/2}$ vs. $h\upsilon$. Extrapolating the linear portion of the obtained curves to the x-axis, where $\alpha$= 0, gives the ${E}_{{\text{gd}}}$ and ${E}_{{\text{gi}}}$ values. The obtained values are listed in Table 1. The PMMA/PVAc blend and its composites are dual-band gap materials. In region (I), the ${E}_{{\text{gd}}}$ and ${E}_{{\text{gi}}}$ of the PMMA/PVAc blend are 4.2 and 4.05 eV, respectively, decreased to 4.1 and 3.7 eV with increasing LaFeO_3_ loading from 0.5 to 10 wt%. Similarly, In region (II), the ${E}_{{\text{gd}}}$ and ${E}_{{\text{gi}}}$ of the 5.1 and 4.9 eV, decreased to 4.7 and 3.9 eV, respectively, with increasing LaFeO_3_. This dual-band gap effect was also seen in NiO/PVA nanocomposites, where *E*_g_ (II) went up from 5.4 to 5.8 eV and *E*_gd_ (I) went down from 3.8 to 2.8 eV as the NiO ratio went up to 5 wt%^42^. Dual *E*_g_ were also found for an electrolyte made of Eu-doped polyvinyl alcohol (PVA) and polyvinyl oxide (PEO)^43^. The decrease in ${E}_{{\text{gd}}}$ and ${E}_{{\text{gi}}}$ because of loading the LaFeO_3_ NPs shows that the extra fillers make the blend better at conducting electricity by creating a 3D continuous network of conductors and defects that create charge carriers across the blend's band gap.

**Table 1:** Direct (${E}_{{\text{gd}}}$) and indirect (${E}_{{\text{gi}}}$) optical band gap of the films.

Film composition	In region (I)		In region (II)	
	${E}_{{\text{gd}}}$(eV)	${E}_{{\text{gi}}}$(eV)	${E}_{{\text{gd}}}$(eV)	${E}_{{\text{gi}}}$(eV)
PMMA/PVAc	4.23	4.05	5.10	4.90
0.5 wt% LaFeO_3_	4.21	4.00	5.05	4.80
1.0 wt% LaFeO_3_	4.20	3.95	5.00	4.70
3.0 wt% LaFeO_3_	4.17	3.90	4.90	4.60
6.0 wt% LaFeO_3_	4.13	3.80	4.80	4.50
10 wt% LaFeO_3_	4.10	3.70	4.70	4.40

### TGA and DSC analysis

**Figure 9:**
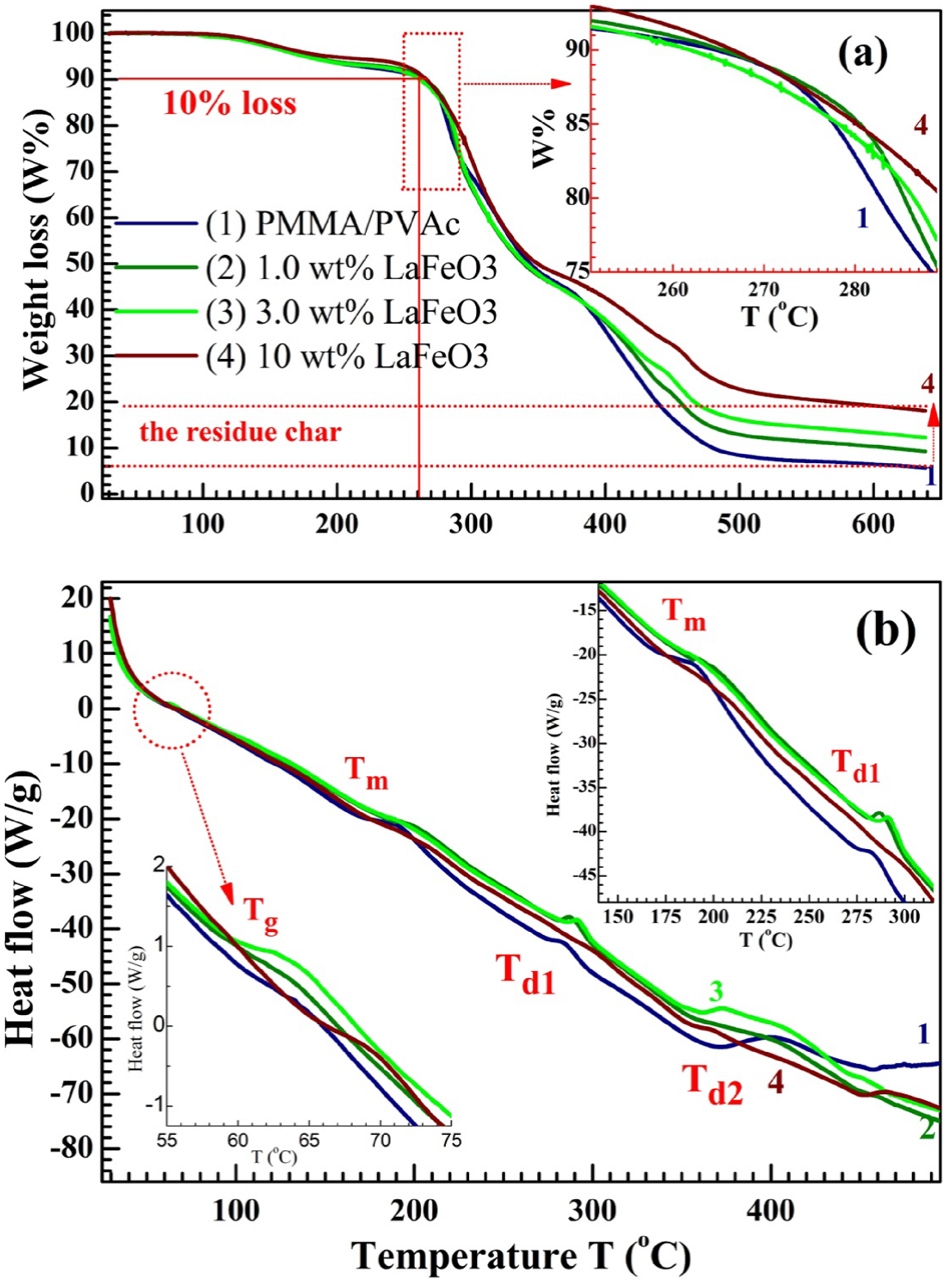
(**a**) TGA and (**b**) DSC thermograms for PMMA/PVAc and blend loaded with 1.0, 3.0 and 10 wt% LaFeO_3_.

TGA and DSC are thermal characterization techniques widely used for exploring the thermal stability and transitions in the materials, under a pre-controlled temperature in an inert atmosphere. The weight loss W (%) of PMMA/PVAc films and heat flow (W/g) are recorded as a function of temperature. The TGA thermograms are shown in Fig. 9a. No W% occurred in the temperature range of 25–130 °C. A first W% (*˂* 10) is seen between 130 and 190 °C. This is because of the spread of persistent THF, moisture, and CO_2_ evaporation^44^. Between 190 and 260 °C, the films show thermal stability, as the W (%) loss is marginal. In other words, the onset temperature for the decomposition of the films is ~ 260 °C. Thus, the blend exhibits higher thermal stability from 25 °C to 260 °C. In addition, the inset of this figure illustrates an improvement in the thermal stability of 3.0 and 10 wt% LaFeO_3_/blend compared to the pure blend. In the ranges of 260–340 °C and 380–480 °C, the films undergo the second and third (final) decompositions. These decompositions are attributed to the degradation (molecular weight reduction) of the blend chains and the degradation of unsaturated bonds in the blend^45,46^. W% after the 2nd stage of degradation is 50%. At the temperature range of 490–640 °C, the remaining weight of the residue char for the PMMA/PVAc blend is ~ 8%, increased to about 20% for the heavily doped film (10 W% LaFeO_3_/blend).

Figure 9b shows the DSC data for the films. Four endothermic peaks can be noticed. The first one is small and located in the temperature range of 58–72 °C (see the inset of the figure) and is owing to the glass–rubber transition (*T*_g_), arising due to the micro-Brownian segmental motion of main chains. The 2^ed^ endothermic peak is at about *T*_m_ = 160–190 °C, and is attributed to the melting of the blends. Loading LaFeO_3_ decreases *T*_g_ from 61 to 59, indicating the flexibility improvement of the blend, *i.e.,* leads to less rigid segments. However, loading the fillers at 10 wt% ratios increased *T*_g_. The *T*_m_ improved to 165 to 171 °C with an increasing filler ratio. The 3^rd^ and 4^th^ endothermic peaks are in the range of 270–290 °C and 350–360 °C and they are attributed to the decomposition of the blends. The values of *T*_g_, *T*_m_, *T*_d1_, and *T*_d2_ are listed in Table 2. The DSC curves of PMMA/PVAc display a single *T*_g_. This indicates the miscibility of the two polymers in the blend, which is consistent with the SEM observation and FTIR spectra. The *T*_d1_ values go up when the LaFeO_3_ ratio goes up, which proves that the PMMA/PVAc blend is more stable at high temperatures^45^. Loading NiO NPs with a content ˃ 2.0 wt% inside the PMMA/PVC blend increased the Tg and Tm of the blend. This is owing to the NiO NPs agglomeration and the increased crystallinity^47^. Our TGA and DSC results show some improvements in the blend thermal properties and stability after loading LaFeO_3_ NPs and these nanocomposites can be utilized efficaciously in the microelectronic industry and space applications at temperatures up to 260 °C.

**Table 2:** The *T*_g_, *T*_m_, *T*_d1_, and *T*_d2_ values of the films.

Film composition	${T}_{g}$(°C)	${T}_{m}$(°C)	${T}_{d1}$(°C)	${T}_{d2}$(°C)
PMMA/PVAc	61	165	275	360
1.0 wt% LaFeO_3_	60	169	279	361
3.0 wt% LaFeO_3_	59	173	281	357
10 wt% LaFeO_3_	64	171	289	352

### Radiation shielding features

**Figure 10:**
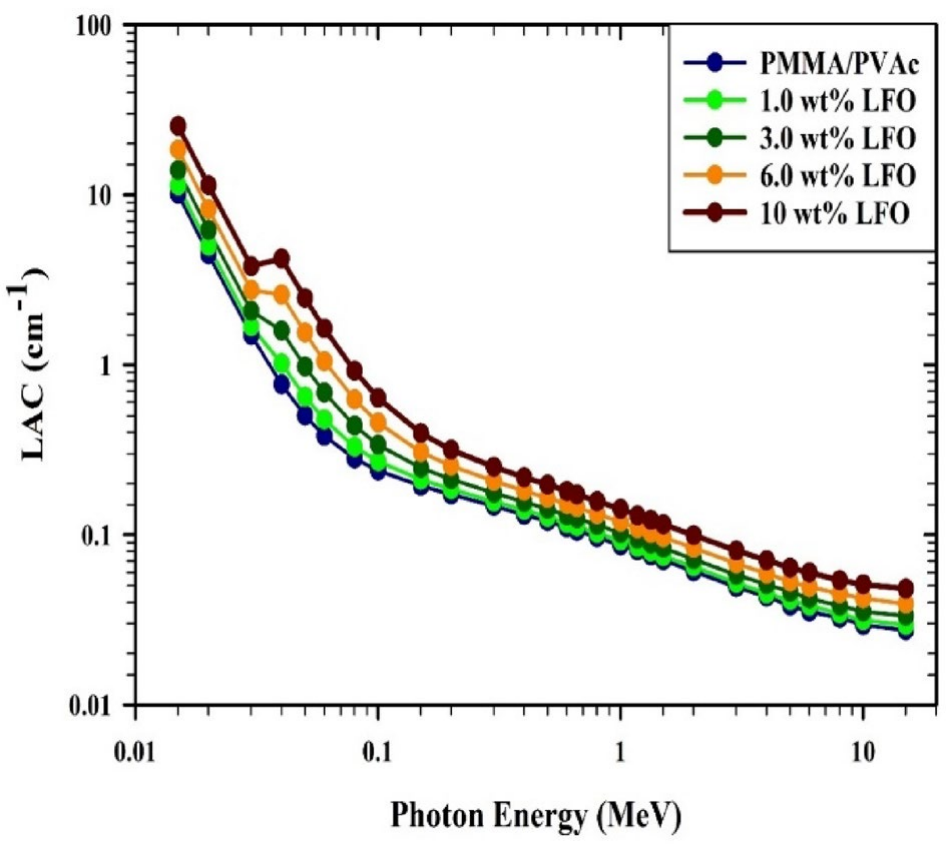
The variation of LAC for PMMA/PVAc blend with various LaFeO_3_ concentrations.

**Figure 11:**
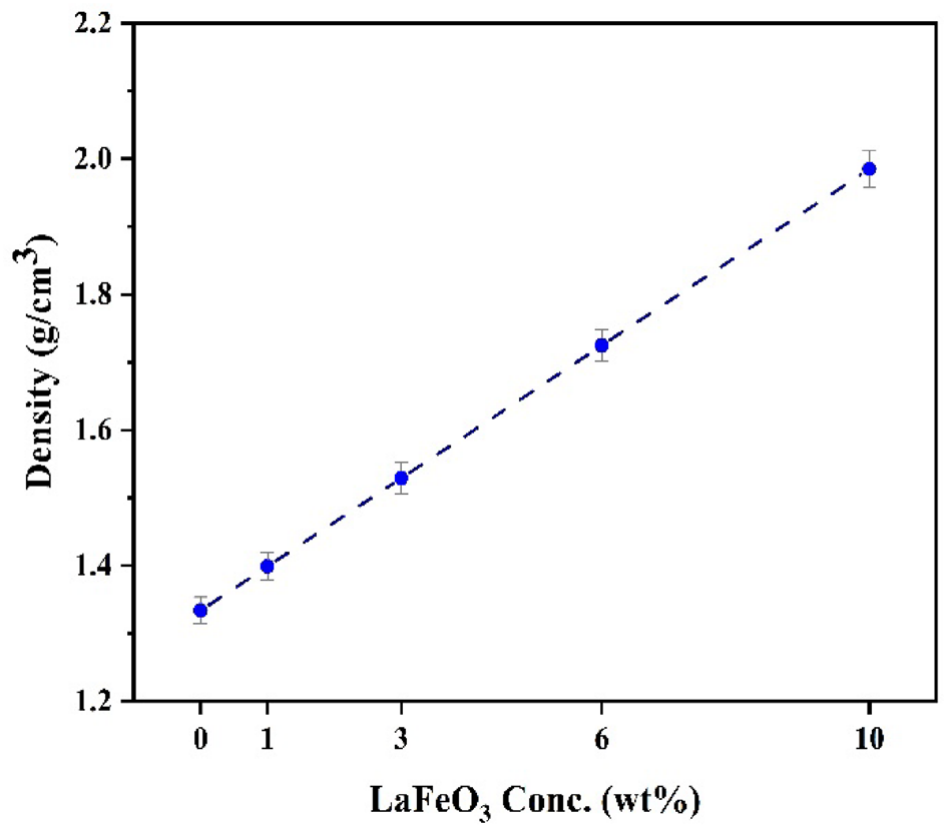
The density values of PMMA/PVAc + x% LaFeO_3_ composite films.

The gamma-ray attenuation properties of films that are made of PMMA/PVAc + x% LaFeO_3_ with energies between 0.015 and 15 MeV have been calculated using Phy-X/PSD^28^. Figure 10 displays how the linear attenuation coefficient (LAC) values change for PMMA/PVAc composite films when photon energy is present. At low energies (*E* < 0.5 MeV), the photoelectric effect (PE) interaction is very strong. The absorption cross-section is related to the atomic number (*Z*^4–5^) and inversely proportional to the photon energy (E^(7/2)^)^31^. The PMMA/PVAc blend composite film has the lowest LAC values, whereas the 10 wt% LaFeO_3_ composite film has the most significant. The difference between the LAC values of PMMA/PVAc + x% LaFeO_3_ composite films gets smaller as the photon energy goes up. Within the photon energy domain of 0.5–1.5 MeV, the Compton scattering (CS) interaction has a strong influence, which is responsible for this occurrence^30^. There is a perfect match between the atomic number (*Z*) and photon energy (*E*) in the PMMA/PVAc + x% LaFeO_3_ composite films when it comes to the absorption cross-section. At photon energies above 1.0 MeV, the pair production (PP) interaction predominates, indicating that there is a proportional association between the interaction cross-section and both (Z^2^) and (log *E*)^48^. Additionally, the *M*_W_ and $\rho$ of the PMMA/PVAc + x% LaFeO_3_ composite films have significantly enhanced and had an impact on gamma-ray absorption due to the addition of components such as metal or metal oxides, which have a high atomic number, *M*_W_, and $\rho$^49,50^. As the LaFeO_3_ increases, the examined PMMA/PVAc + x% LaFeO_3_ composite films become denser. The difference in the density ($\rho )$ between LaFeO_3_, which has $\rho$ = 6.51 gcm^-3^, and PMMA/PVAc, which has $\rho$ = 1.334 gcm^-3^, may be the cause of the observed increase in $\rho$ of PMMA/PVAc + x% LaFeO_3_ composite films. PMMA/PVAc composite films with x% LaFeO_3_ had $\rho$ in the range of 1.334–1.985 gcm^-3^, as shown in Fig. 11.

**Figure 12:**
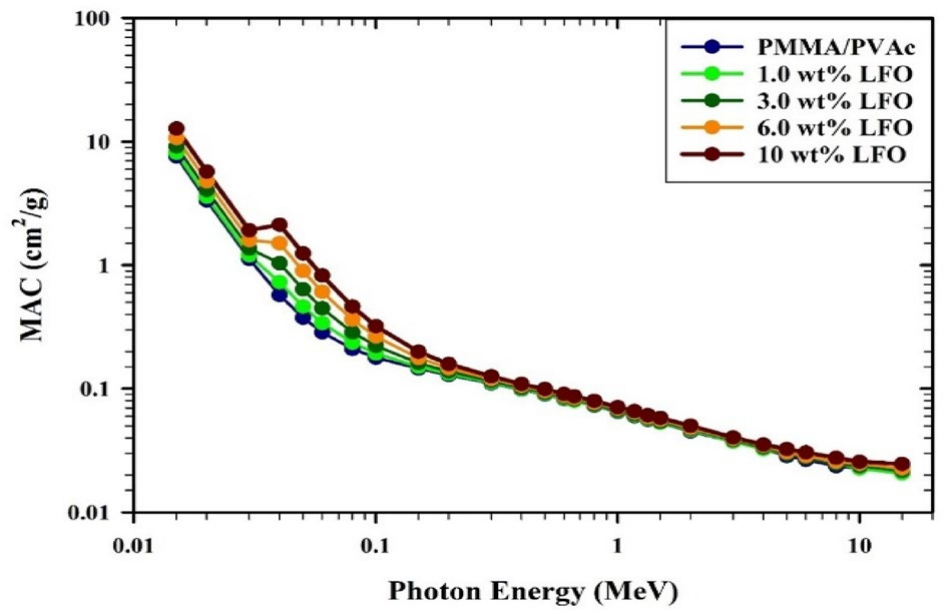
The variation of MAC for PMMA/PVAc + x% LaFeO_3_ films.

Figure 12 displays the MAC (given by Eq. (2)) of PMMA/PVAc + x% LaFeO_3_ composite films. The MAC values progressively decrease as the photon energy grows. The cause of this behavior is the photoelectric effect in the energy range (E < 0.5 MeV). The Compton scattering interaction is to blame for the MAC values changing quite slowly as the photon energy increases up to 0.5 MeV. The MAC results improve as the LaFeO_3_ concentration increases. A comparison of the mass attenuation coefficient values produced using the Phy-X/PSD program with those acquired from the XCOM program^51^ is shown in Table 3. The MAC results of PMMA/PVAc + x% LaFeO_3_ composite films typically increase with increasing LaFeO_3_ content from 0 to 10% wt and decrease with increasing photon energy. Furthermore, a strong correlation was found between the Phy-X/PSD values and the XCOM results. The MAC values for the PMMA/PVAc + 10% LaFeO_3_ composite film were enhanced to 0.0883 cm^2^g^-1^ at 0.662 MeV in comparison to PMMA/PVAc bland (MAC = 0.0806 cm^2^g^−1^). This identified modification could have been caused by the high-density LaFeO_3_ component added to the PMMA/PVAc system. The produced PMMA/PVAc + x% LaFeO_3_ composite films exhibit improved shielding properties as a result of the addition of additional LaFeO_3_ to the PMMA/PVAc matrix.

**Table 3:** The mass attenuation coefficients for PMMA/PVAc + x% LaFeO_3_ (LFO) composite films.

Photon energy (MeV)	Mass attenuation coefficient (cm^2^/g)									
	PMMA/PVCAc		1%LFO		3%LFO		6%LFO		10%LFO	
	XCOM	Phy	XCOM	Phy	XCOM	Phy	XCOM	Phy	XCOM	Phy
0.015	7.6453	7.6455	8.1655	8.1657	9.2059	9.2060	10.7665	10.7665	12.8473	12.8471
0.02	3.3767	3.3764	3.6159	3.6157	4.0943	4.0942	4.8119	4.8119	5.7687	5.7689
0.03	1.1354	1.1353	1.2151	1.2151	1.3747	1.3747	1.6140	1.6140	1.9332	1.9331
0.04	0.5814	0.5815	0.7378	0.7379	1.0506	1.0507	1.5198	1.5199	2.1454	2.1455
0.05	0.3814	0.3814	0.4691	0.4691	0.6446	0.6446	0.9078	0.9078	1.2588	1.2587
0.06	0.2904	0.2904	0.3448	0.3448	0.4537	0.4537	0.6169	0.6170	0.8346	0.8347
0.08	0.2134	0.2134	0.2390	0.2390	0.2902	0.2902	0.3670	0.3670	0.4694	0.4694
0.1	0.1813	0.1813	0.1957	0.1957	0.2246	0.2246	0.2678	0.2678	0.3254	0.3254
0.15	0.1477	0.1477	0.1531	0.1531	0.1640	0.1640	0.1803	0.1803	0.2021	0.2020
0.2	0.1314	0.1314	0.1344	0.1344	0.1404	0.1404	0.1494	0.1495	0.1615	0.1615
0.3	0.1123	0.1122	0.1138	0.1138	0.1170	0.1170	0.1218	0.1218	0.1281	0.1281
0.4	0.1000	0.1000	0.1011	0.1011	0.1034	0.1034	0.1069	0.1069	0.1115	0.1115
0.5	0.0911	0.0911	0.0920	0.0920	0.0939	0.0939	0.0968	0.0968	0.1006	0.1006
0.6	0.0842	0.0842	0.0850	0.0850	0.0866	0.0866	0.0891	0.0891	0.0924	0.0924
0.6617	0.0806	0.0806	0.0813	0.0813	0.0829	0.0829	0.0852	0.0852	0.0883	0.0883
0.8	0.0738	0.0738	0.0745	0.0745	0.0759	0.0759	0.0779	0.0779	0.0807	0.0807
1	0.0663	0.0663	0.0669	0.0669	0.0681	0.0681	0.0699	0.0699	0.0723	0.0723
1.173	0.0613	0.0613	0.0618	0.0618	0.0629	0.0629	0.0645	0.0646	0.0667	0.0667
1.333	0.0574	0.0574	0.0579	0.0579	0.0589	0.0589	0.0605	0.0605	0.0625	0.0625
1.5	0.0540	0.0540	0.0545	0.0545	0.0555	0.0555	0.0569	0.0569	0.0588	0.0588
2	0.0465	0.0465	0.0469	0.0469	0.0478	0.0478	0.0490	0.0490	0.0507	0.0507
3	0.0377	0.0377	0.0381	0.0381	0.0388	0.0388	0.0399	0.0399	0.0414	0.0414
4	0.0327	0.0327	0.0330	0.0330	0.0337	0.0337	0.0348	0.0348	0.0361	0.0361
5	0.0295	0.0295	0.0298	0.0298	0.0305	0.0305	0.0315	0.0315	0.0328	0.0328
6	0.0273	0.0273	0.0276	0.0276	0.0283	0.0283	0.0293	0.0293	0.0306	0.0306
8	0.0245	0.0245	0.0248	0.0248	0.0255	0.0255	0.0265	0.0265	0.0278	0.0278
10	0.0228	0.0228	0.0232	0.0232	0.0238	0.0238	0.0249	0.0249	0.0263	0.0263
15	0.0208	0.0208	0.0212	0.0212	0.0219	0.0219	0.0230	0.0230	0.0245	0.0245

**Figure 13:**
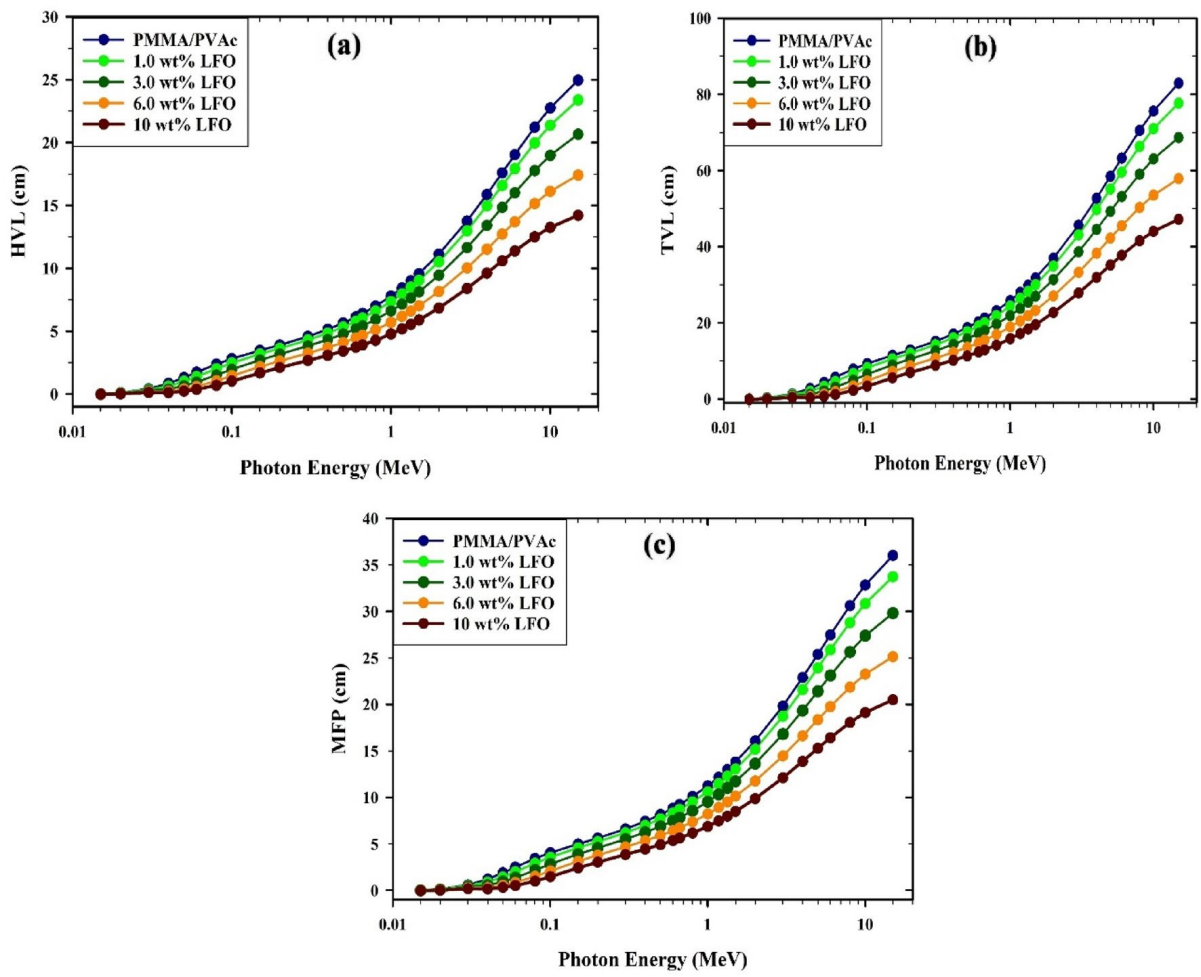
The varying values of (**a**) half-value layer (HVL), (**b**) tenth-value layer (TVL), and (**c**) mean free path (MFP) for PMMA/PVAc + x% LaFeO_3_ composite films.

Investigations of the HVL, TVL, and MFP (given by Eqs. (3), (4), (5) can be used to describe a substance's ability to absorb radiation. They are indicators of improved radiation absorption capability when their values are reduced. Figure 13a shows the fluctuation of HVL values with photon energy for the PMMA/PVAc + x% LaFeO_3_ composite films. It has been discovered that as photon energy rises in the 0.015–1 MeV region, the HVL values improve. HVL values between 0.015 and 0.1 MeV do not go above 1cm. The PMMA/PVAc + 10% LaFeO_3_ composite films with the greatest LaFeO_3_ concentration exhibited the smallest HVL value when compared to the other composite films examined. The HVL data of PMMA/PVAc + x% LaFeO_3_ composite films were documented at 1.332 MeV (0.662 MeV), with 9.053 (6.450), 8.556 (6.092), 7.693 (5.470), 6.646 (4.716), and 5.587(3.955) cm for x = 0, 1, 3, 6, and 10%, respectively. This makes it more successful at blocking radiation. Figure 13b shows a similar trend for TVL values grow as the input photon's energy rises to 1.5 MeV and that the link dissipates after this point. TVL values decrease when the LaFeO_3_ proportion increases (from 0 to 10%). According to the results in Fig. 13c, the Mean Free Path (MFP) values show a dependency on photon energy up to a point around 1.5 MeV, after which their correlation ends. The protective ability of PMMA/PVAc films against gamma radiation rises with the addition of LaFeO_3_ to PMMA/PVAc (0–10%), associated with a decrease in mean free path values.

**Figure 14:**
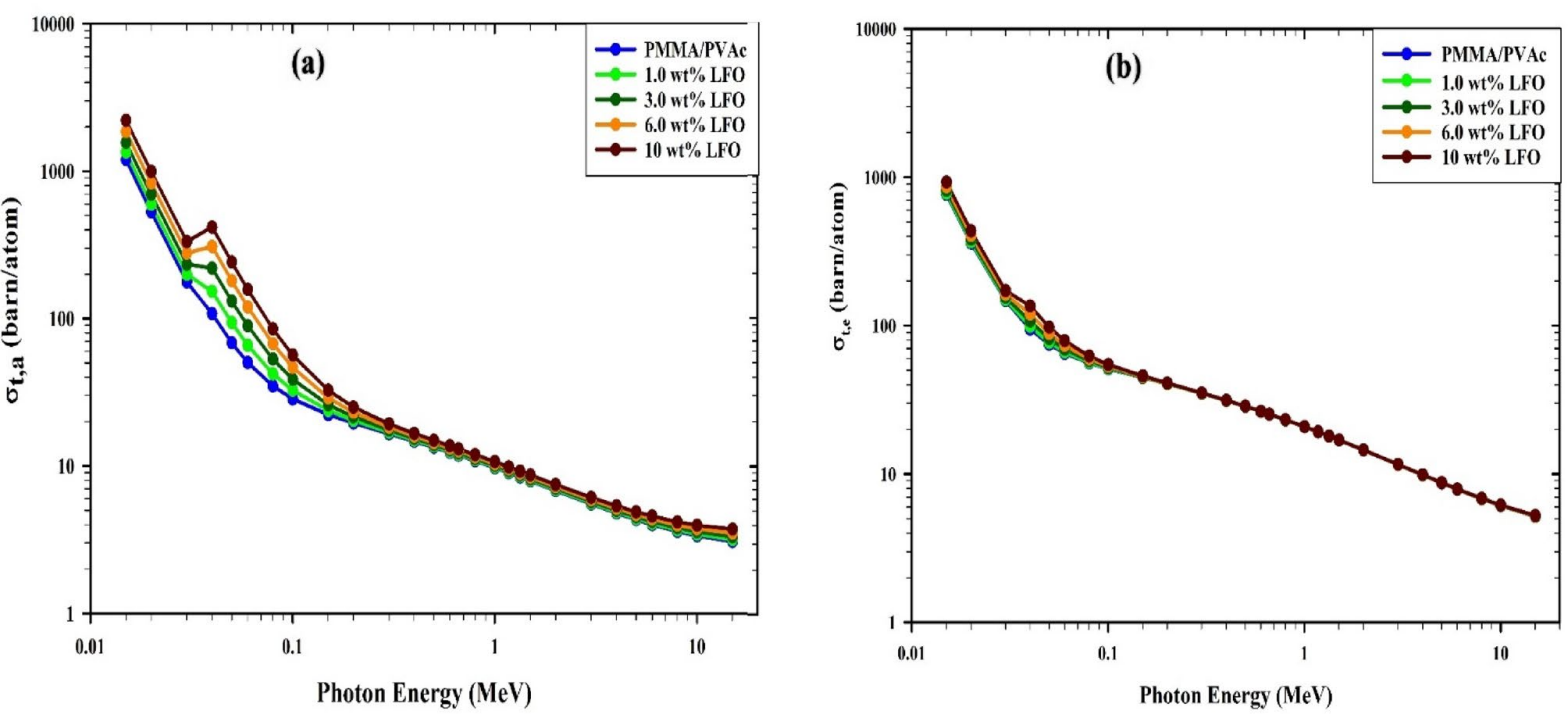
Variation (**a**) the total atomic cross section (σ_t, a_), (**b**) the total electronic cross section (σ_t, e_) versus the photon energy for PMMA/PVAc + x% LaFeO_3_ composite films.

Figure 14a displays the variation of the σ_t,a_ (Eq. (6)) for PMMA/PVAc + x% LaFeO_3_ composite films with the photon energy. The σ_t,a_ significantly decreases as energy levels rise. These observable variations are due to the photo-electric atomic cross sections (σ_photo_) at lower energies and the Compton scattering (σ_Compton_) at higher energies. The prepared PMMA/PVAc + x% LaFeO_3_ composite films' σ_t,e_ (Eq. (7)) shows the same behavior as the (σ_t,a_) and the MAC in Fig. 14b, *i.e.*, a reduction with increasing photon energy and a rise with increasing LaFeO_3_ content^31,52^.

**Figure 15:**
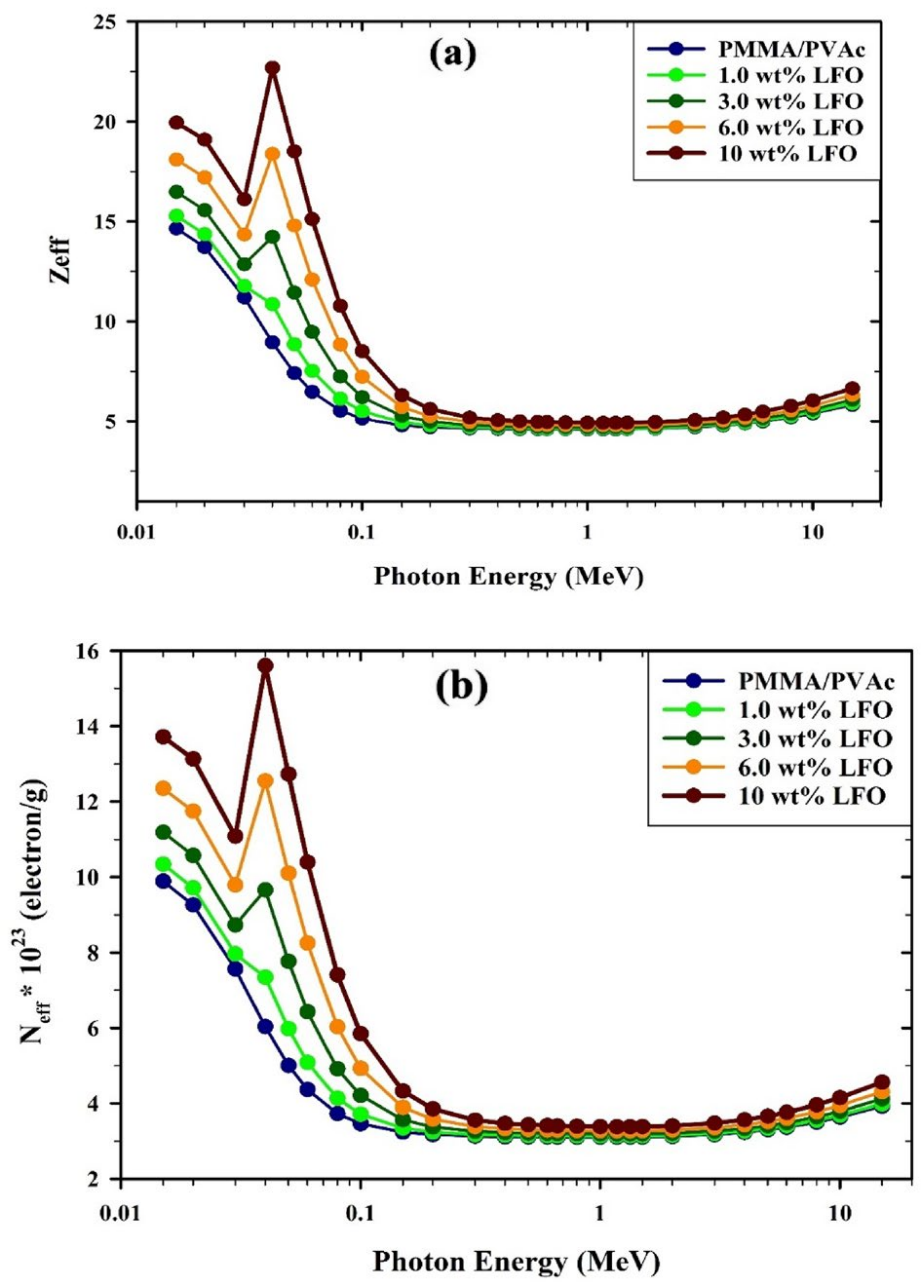
(**a**) the effective atomic number (Z_eff_) and (**b**) effective electron density (N_eff_) for PMMA/PVAc + x% LaFeO_3_ composite films.

The *Z*_eff_ (Eq. (8)) of the manufactured PMMA/PVAc composite films varies significantly depending on the various materials incorporated into them. As seen in Fig. 14a, adding LaFeO_3_ to the PMMA/PVAc polymer causes an increase in the *Z*_eff_ of the PMMA/PVAc + x% LaFeO_3_ composite films. The interaction probabilities of the photoelectric effect, the Compton scattering effect, and the pair creation process were proportional to (*Z*_eff_)^4–5^, (*Z*_eff_), and (*Z*_eff_)^2^, respectively. As seen in Fig. 15a, the incorporation of elements with relatively high atomic numbers (*Z* = 26 for Fe and *Z* = 57 for La) causes a rise in *Z*_eff_ as the concentration of LaFeO3 increases. The energy dependency of *Z*_eff_ likewise displays MAC-similar behavior^53^. The relationship between the LaFeO_3_ concentration and the *N*_eff_ (Eq. (9)) for PMMA/PVAc + x% LaFeO_3_ composite films is shown in Fig. 15b. N_eff_ also responded to the increase in LaFeO_3_ concentration similarly to *Z*_eff_. The composite film made of PMMA/PVAc with 10% LaFeO_3_ exhibits a fairly constant state of equilibrium. The PMMA/PVAc + x% LaFeO_3_ composite films' behavior in *N*_eff_ can be linked to their constituent polymers' comparable atomic numbers, particularly those of the C, H, and O atoms^54^.

## Conclusion

We have successfully prepared LaFeO_3_ NPs and LaFeO_3_/PMMA/PVAc nanocomposite films by facile chemical methods. XRD and HR-TEM showed the high purity and good crystallinity of LaFeO_3_ that has a particle size of 79 nm, and several diffraction peaks of LaFeO_3_ appeared in XRD patterns of the semi-crystalline PMMA/PVAc blend. FTIR revealed the formation of Fe–O–Fe and La–O–La at 548, 920, and 970 cm^–1^, and all reactive functional groups of the blend existed. Increasing the LaFeO_3_ content ratio from 0.5 to 6.0 wt% led to a significant decrease in the absorption band intensities. FE-SEM showed the homogeneity good dispersion and uniform distribution of the fillers on the film surface and inside the blend. The elemental mapping confirmed the uniform distribution of La and Fe atoms event at the highest doping ratio (10 wt%). The blend displayed transmittance ranged from 30 to 74% in the visible and IR regions. Increasing LaFeO_3_ ratios made *k* increases linearly with the wavelength. Tauc's method showed that the LaFeO_3_/PMMA/PVAc films exhibit dual direct and indirect band gaps on the low energy region (${E}_{{\text{gd}}}$ = 4.1–4.23), (${E}_{{\text{gi}}}$ = 3.7–4.05) and on high energy region (${E}_{{\text{gd}}}$ = 4.7–5.1), (${E}_{{\text{gi}}}$ = 4.4–4.9). The optical properties of the films suggest the utilization of the materials for a wide range of applications such as optoelectronic devices and sensors. TGA showed an improvement in the thermal properties and stability of the samples from RT to 260 °C, with increasing LaFeO_3_ content. In addition, LaFeO_3_/PMMA/PVAc has *T*_g_ in the range of 59–64 °C, *T*_m_ in the range of 165–173 °C, and decomposes at two different stages, where *T*_d1_ and *T*_d2_ are in the range of (275–289 °C and 352–361 °C, respectively. The improvement in the thermal properties makes these samples suitable for the microelectronic industry and space applications at temperatures up to 260 °C. This study introduces novel LaFeO_3_/PMMA/PVAc composite films (0, 1, 3, 6, and 10 wt%) as flexible and sustainable radiation shielding materials. LaFeO_3_ was implemented to improve the different density and attenuation characteristics of PMMA/PVAc. In the investigation, Phy-X/PSD and XCOM were used to compare the mass attenuation coefficient (MAC) of composite films made of PMMA/PVAc and x% LaFeO_3_. The inquiry looked into the relationship between *Z*_eff_ and *N*_eff_ for composite films and the LaFeO_3_ content. Ultimately, the radiation-attenuating properties of PMMA/PVAc + x% LaFeO_3_ composite films provide them with excellent materials for radiation-blocking purposes.

## Data Availability

The authors declare that the data supporting the findings of this study are available within the article.
